# Supplementary material for: Novel temporal and spatial patterns of metastatic colonization from breast cancer rapid-autopsy tumor biopsies
Source: Genome Med. 2021 Oct 28;13:170. doi: 10.1186/s13073-021-00989-6 (PMC8555066; doi:10.1186/s13073-021-00989-6)
Supplement: Supplementary file 1 — Additional file 1: Supplementary Figures S1-S18. [file 13073_2021_989_MOESM1_ESM.pdf]

Fig. S1

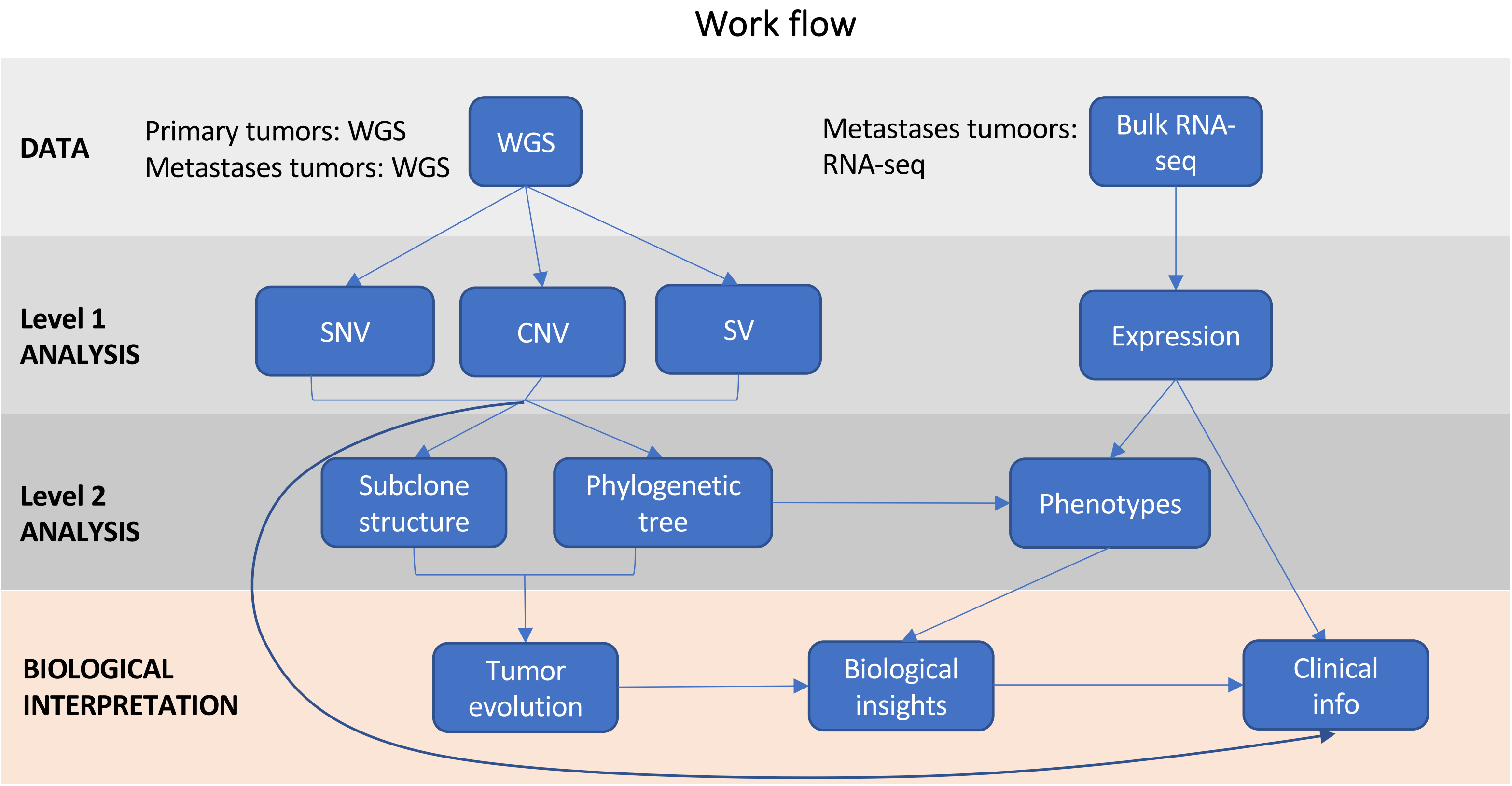
Work flow
DATA
Primary tumors: WGS
Metastases tumors: WGS
WGS
Metastases tumoors:
RNA-seq
Bulk RNA-seq
Level 1
ANALYSIS
SNV
CNV
SV
Expression
Level 2
ANALYSIS
Subclone structure
Phylogenetic tree
Phenotypes
BIOLOGICAL
INTERPRETATION
Tumor evolution
Biological insights
Clinical info

Fig. S1. Overview of the workflow for this study.

Fig. S2

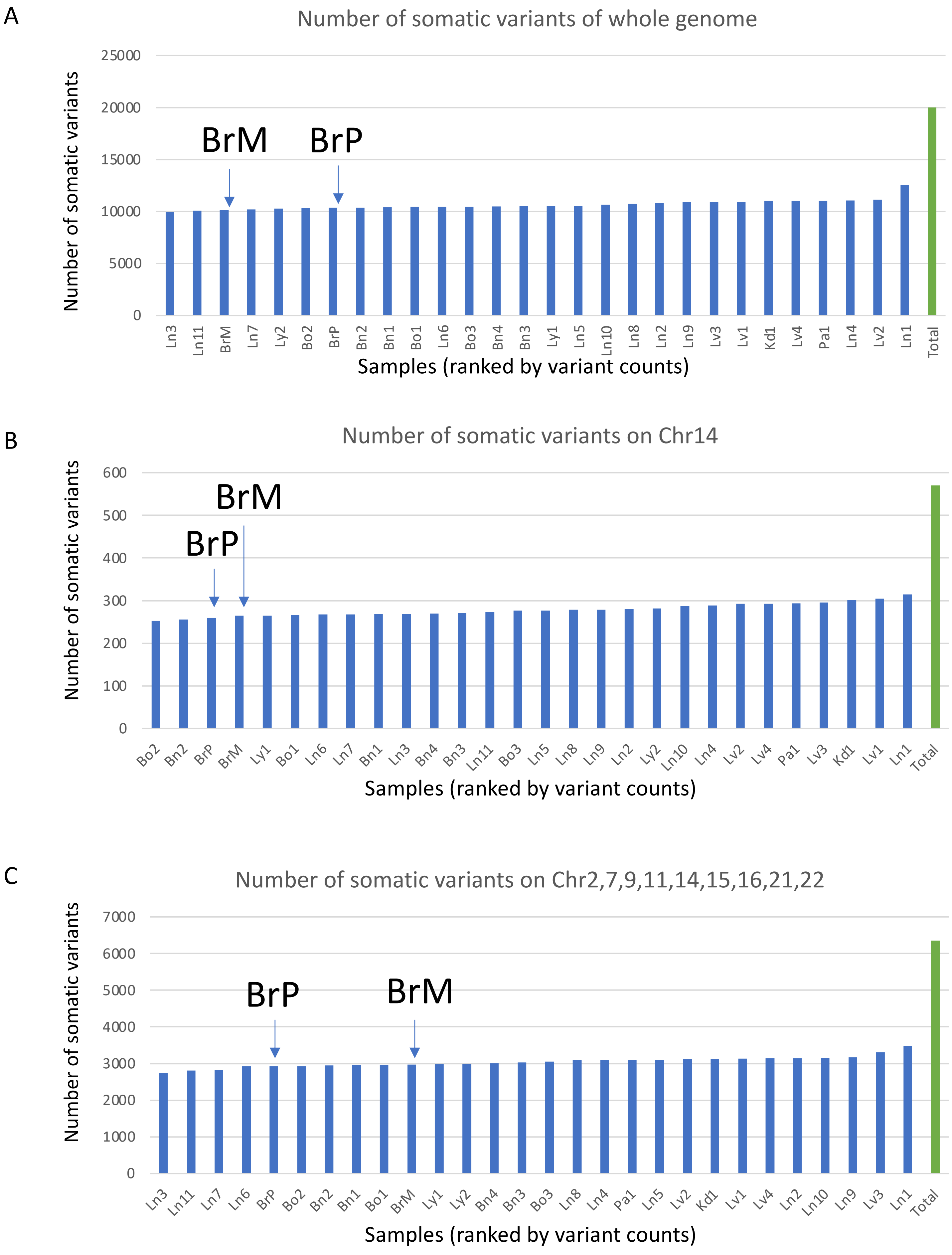

A
Number of somatic variants of whole genome
Number of somatic variants
25000
20000
15000
10000
5000
0
BrM
BrP
Ln3 Ln11 BrM Ln7 Ly2 Bo2 BrP Bn2 Bn1 Bo1 Ln6 Bo3 Bn4 Bn3 Ly1 Ln5 Ln10 Ln8 Ln2 Ln9 Lv3 Lv1 Kd1 Lv4 Pa1 Ln4 Lv2 Ln1 Total
Samples (ranked by variant counts)
B
Number of somatic variants on Chr14
Number of somatic variants
600
500
400
300
200
100
0
BrM
BrP
Bo2 Bn2 BrP BrM Ly1 Bo1 Ln6 Ln7 Bn1 Ln3 Bn4 Bn3 Ln11 Bo3 Ln5 Ln8 Ln9 Ln2 Ly2 Ln10 Ln4 Lv2 Lv4 Pa1 Lv3 Kd1 Lv1 Ln1 Total
Samples (ranked by variant counts)
C
Number of somatic variants on Chr2,7,9,11,14,15,16,21,22
Number of somatic variants
7000
6000
5000
4000
3000
2000
1000
0
BrP
BrM
Ln3 Ln11 Ln7 Ln6 BrP Bo2 Bn2 Bn1 Bo1 BrM Ly1 Ly2 Bn4 Bn3 Bo3 Ln8 Ln4 Pa1 Ln5 Lv2 Kd1 Lv1 Lv4 Ln2 Ln10 Ln9 Lv3 Ln1 Total
Samples (ranked by variant counts)

Fig. S2. Somatic variant counts across all tumor samples. (A) Whole genome somatic variant counts for all tumor samples. **(B)** Somatic variant counts on chromosome 14. **(C)** Somatic variant counts on chromosome 2,7,9,11,14,15,16,21,22. X-axis: samples ranked by variant counts from smallest to largest. Y-axis: the number of somatic variants. Green bars represent the total number of variants. BrP and BrM, which were sequenced at 45X coverage, had similar mutation counts as other rapid-autopsy samples sequenced at 60X coverage.

Fig. S3

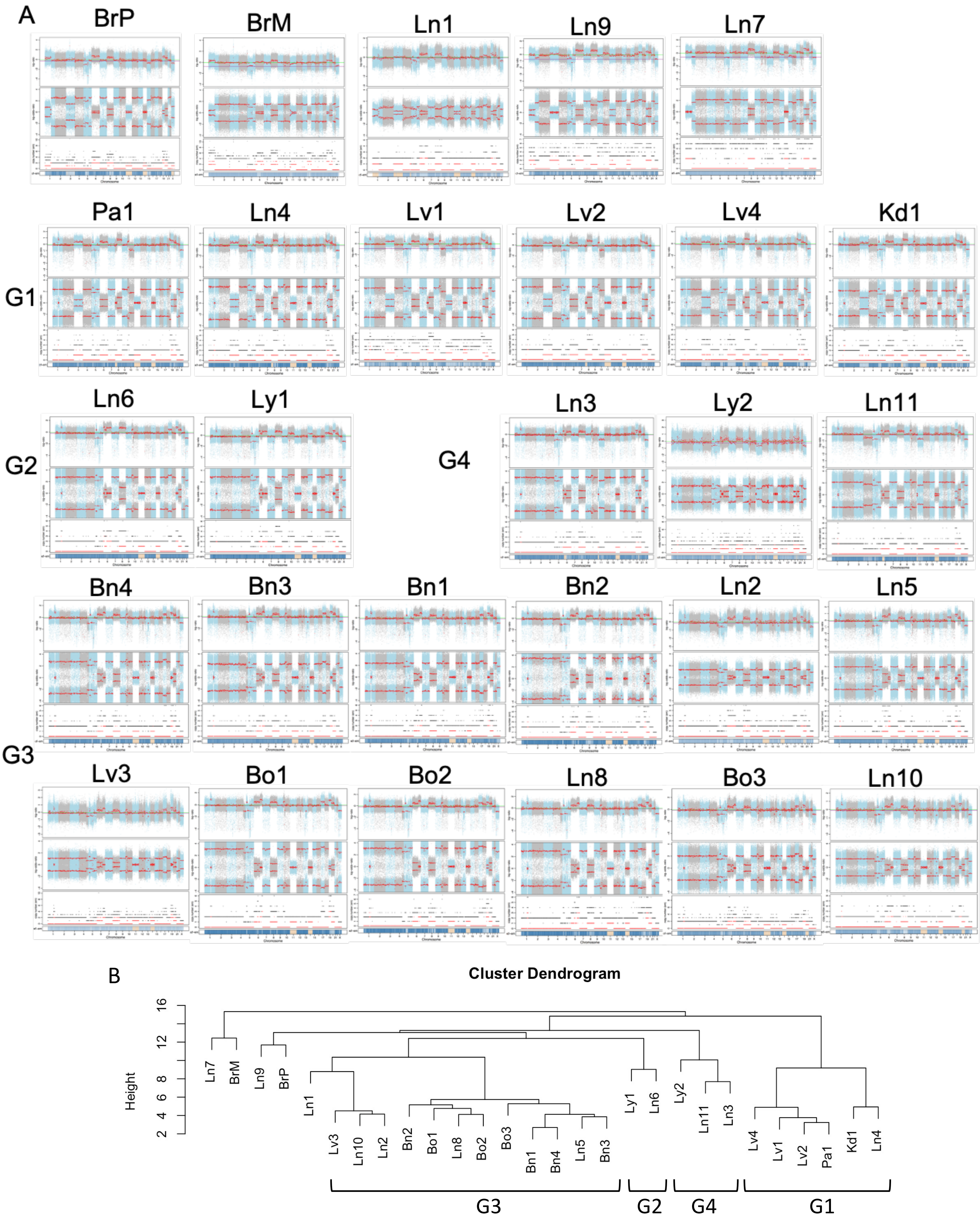
A
BrP
BrM
Ln1
Ln9
Ln7
Pa1
Ln4
Lv1
Lv2
Lv4
Kd1
G1
Ln6
Ly1
G2
G4
Ln3
Ly2
Ln11
Bn4
Bn3
Bn1
Bn2
Ln2
Ln5
G3
Lv3
Bo1
Bo2
Ln8
Bo3
Ln10
B
Cluster Dendrogram
Height
2 4 6 8 12 16
Ln7 BrM Ln9 BrP Ln1 Lv3 Ln10 Ln2 Bn2 Bo1 Ln8 Bo2 Bo3 Bn1 Bn4 Ln5 Bn3 Ly1 Ln6 Ly2 Ln11 Ln3 Lv4 Lv1 Lv2 Pa1 Kd1 Ln4
G3
G2
G4
G1

Fig. S3. CNV profiles of all samples. (A) CNV profile generated from FACETS. For each sample, top panel is the log2 ratio of tumor copy number to normal copy number. The middle panel is allele-specific log-odds-ratio with chromosomes alternating in blue and grey. The bottom panel is the corresponding integer (total, minor) copy number calls by FACETS. **(B)** Automated sample clustering based on copy number similarity between samples.

Fig. S4

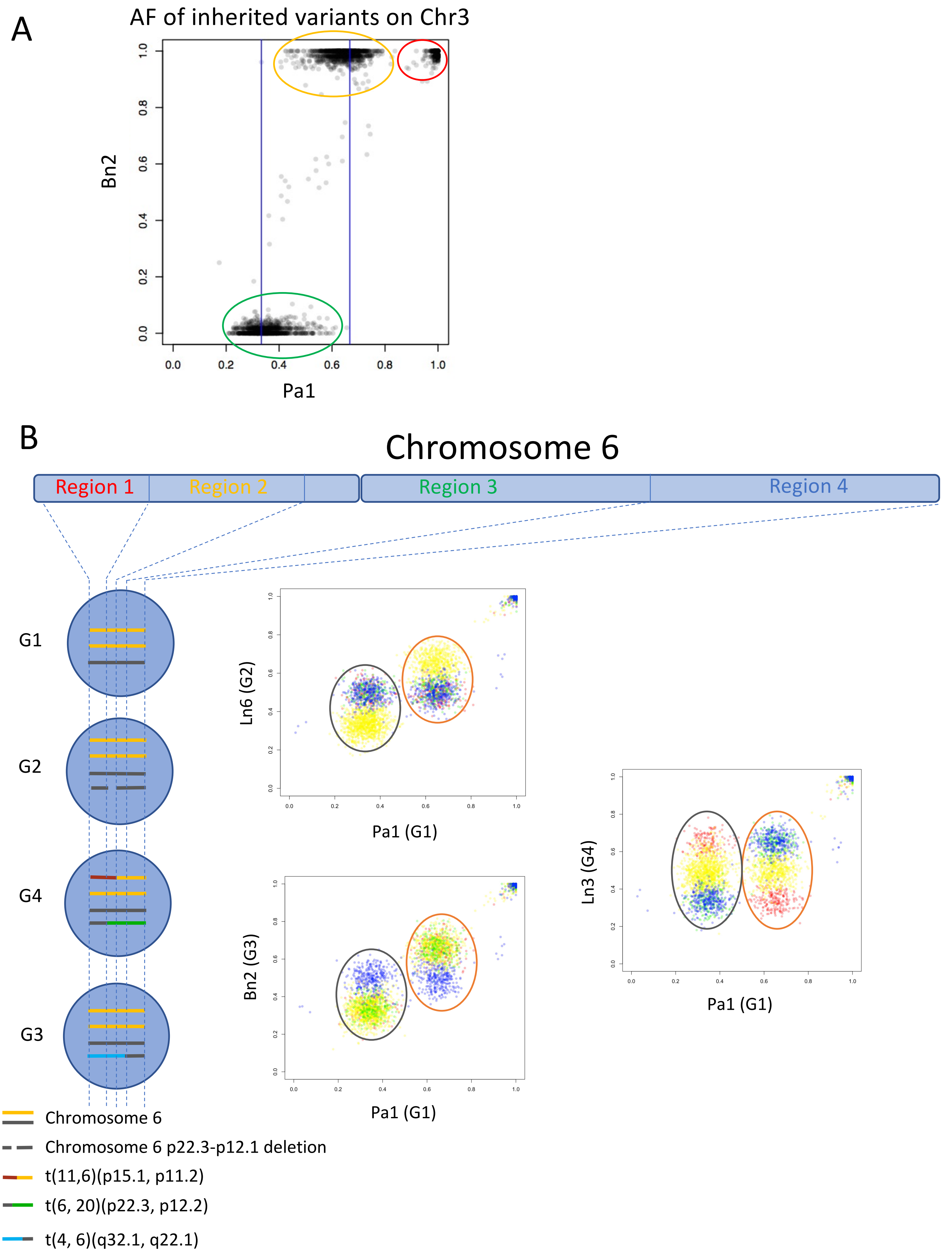
A
AF of inherited variants on Chr3
Bn2
Pa1
0.0
0.2
0.4
0.6
0.8
1.0
B
Chromosome 6
Region 1
Region 2
Region 3
Region 4
G1
G2
G4
G3
Ln6 (G2)
Pa1 (G1)
Bn2 (G3)
Pa1 (G1)
Ln3 (G4)
Pa1 (G1)
Chromosome 6
Chromosome 6 p22.3-p12.1 deletion
t(11,6)(p15.1, p11.2)
t(6, 20)(p22.3, p12.2)
t(4, 6)(q32.1, q22.1)

Fig. S4. Allele specific CNV/LOH call. (A) Scatter plot of allele frequencies of inherited variants on chromosome 3 between sample Pa1 and Bn2. **(B)** Allele frequencies of inherited variants on chromosome 6. Chromosome 6 was divided into four regions based on the breakpoints that caused different structural variants in our samples in different groups. Three scatter plots showed the allele frequencies of inherited variants in different regions between two samples. Each dot represents a variant. Color of the dots matches the region of the variant.

Fig. S5

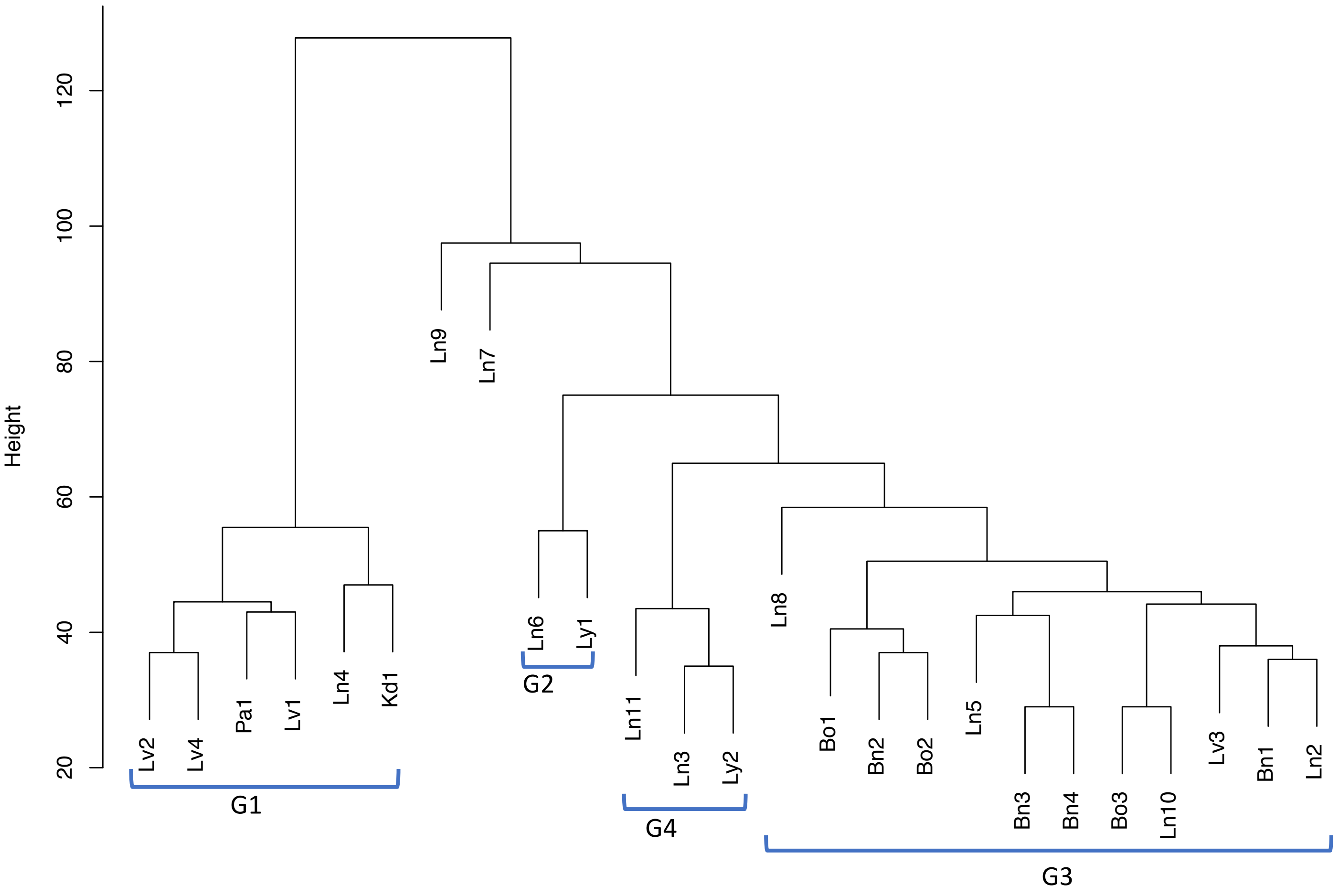
Height
120
100
80
60
40
20
Lv2
Lv4
Pa1
Lv1
Ln4
Kd1
Ln9
Ln7
Ln6
Ly1
Ln11
Ln3
Ly2
Ln8
Bo1
Bn2
Bo2
Ln5
Bn3
Bn4
Bo3
Ln10
Lv3
Bn1
Ln2
G1
G2
G4
G3

Fig. S5. Automated sample clustering based on hamming distance between samples which were encoded as a vector of 0 (absence) or 1 (presence) of somatic short variants on chromosome 14.

Fig. S6

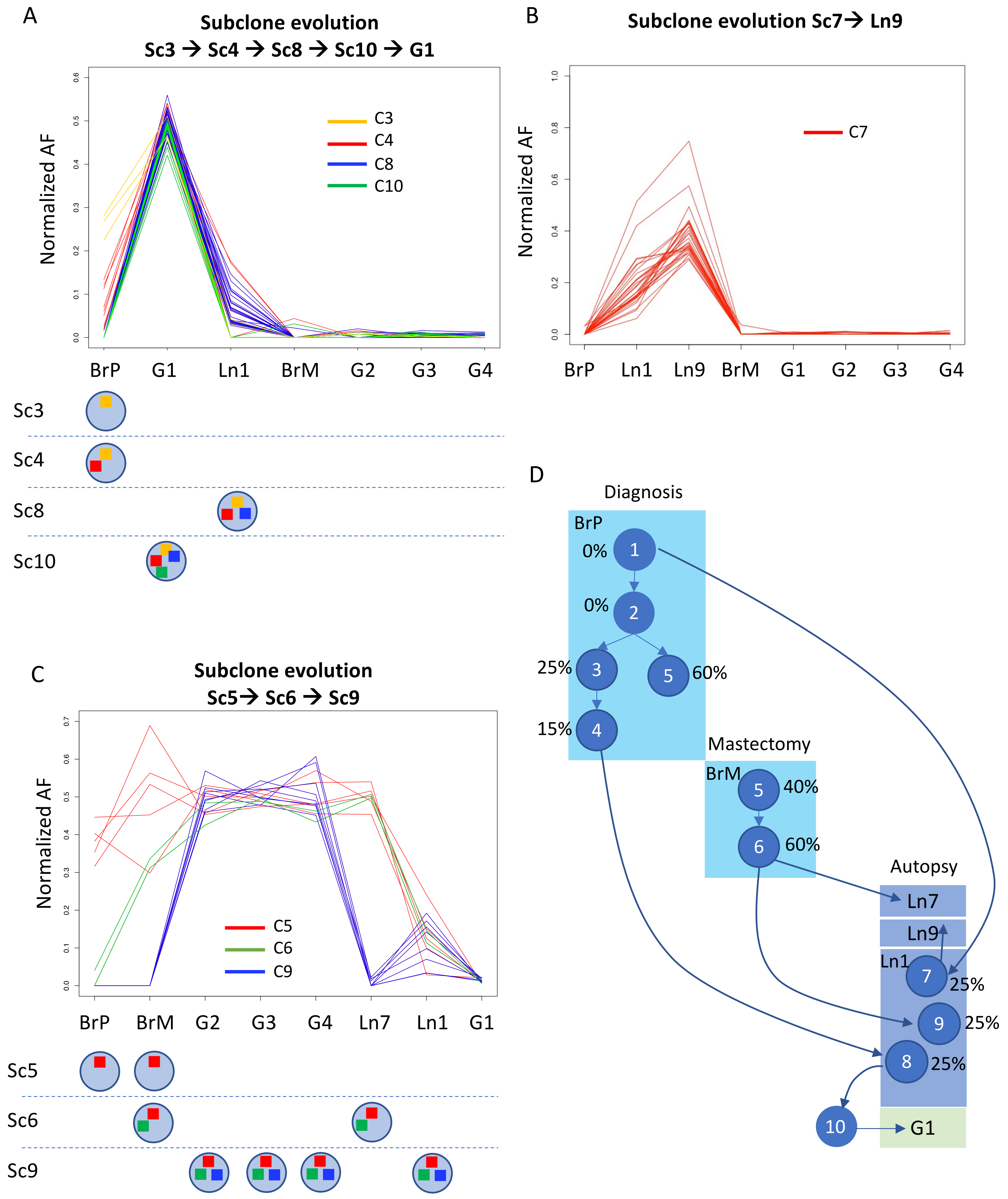
A
Subclone evolution
Sc3 → Sc4 → Sc8 → Sc10 → G1
Normalized AF
C3
C4
C8
C10
BrP
G1
Ln1
BrM
G2
G3
G4
Sc3
Sc4
Sc8
Sc10
B
Subclone evolution Sc7→ Ln9
Normalized AF
C7
BrP
Ln1
Ln9
BrM
G1
G2
G3
G4
C
Subclone evolution
Sc5→ Sc6 → Sc9
Normalized AF
C5
C6
C9
BrP
BrM
G2
G3
G4
Ln7
Ln1
G1
Sc5
Sc6
Sc9
D
Diagnosis
BrP
0%
1
0%
2
25%
3
5
60%
15%
4
Mastectomy
BrM
5
40%
6
60%
Autopsy
Ln7
Ln9
Ln1
7
25%
9
25%
8
25%
10
G1

Fig. S6. Early subclone evolution and expansion in breast tumors and the first invaded lung sites. (A) Subclone evolution Sc3->Sc4->Sc8->Sc10->G1. The top panel is normalized AF of short variants on chromosome 14 clustered into C3, C4, C8, and C10 depicted in yellow, red, blue, and green respectively. The bottom panel is a schematic plot of which subclone contains which variant clusters and which sample contains which subclones. Each row is a subclone. A circle represents a subclone and a colored square inside of the circle represents the corresponding variant cluster in the top panel. For example, Sc3 only contains variants in cluster C3, whereas Sc10 contains variants in C3, C4, C8, and C10. A sample contains subclones below this sample. BrP contains Sc3 and its descendant Sc4. A further evolved subclone Sc8 from Sc4 went to Ln1 and eventually became the common seeder Sc10 for samples in G1. None of these subclones are present in BrM, G2, G3, and G4. **(B)** Subclone evolution Sc7->Ln9. Normalized AF of short variants on chromosome 14 clustered in C7 in red corresponding to new variants appeared in Sc7 shared by only Ln1 and Ln9. **(C)** Subclone evolution Sc5->Sc6->Sc9. The top panel is normalized AF of short variants on chromosome 14 clustered in C5, C6, and C9 depicted in red, green, and blue respectively. The bottom panel showed that BrP contains Sc5 which also appeared in BrM. BrM also contains Sc5's descendant subclone Sc6 which seeded Ln7. Sc9 existed in Ln1 and was the common ancestral clone seeding G2, G3 and G4. **(D)** Estimated CP based on normalized AF of each subclone in BrP, BrM, and Ln1.

Fig. S7

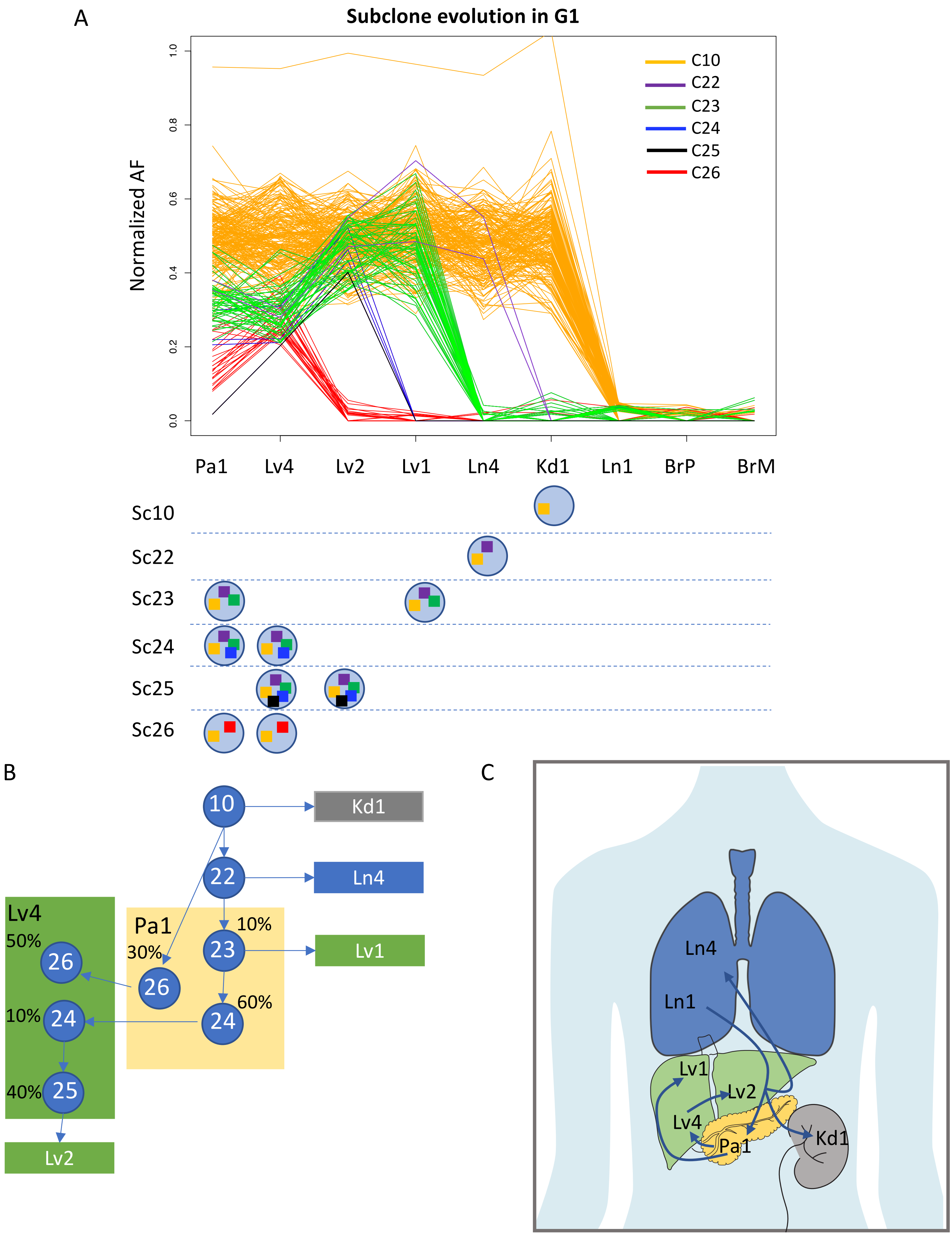
A
Subclone evolution in G1
Normalized AF
0.0
0.2
0.4
0.6
0.8
1.0
C10
C22
C23
C24
C25
C26
Pa1
Lv4
Lv2
Lv1
Ln4
Kd1
Ln1
BrP
BrM
Sc10
Sc22
Sc23
Sc24
Sc25
Sc26
B
10
Kd1
22
Ln4
Lv4
Pa1
10%
23
Lv1
50%
26
30%
26
10%
24
24
60%
40%
25
Lv2
C
Ln4
Ln1
Lv1
Lv2
Lv4
Pa1
Kd1

Fig. S7. Subclone evolution and expansion for samples in G1. (A) The top panel is normalized AF of G1 specific somatic short variants on chromosome 2, 7, 9, 11, 14, 15, 16, 21, and 22 clustered into C10, C22, C23, C24, C25, and C26 depicted in orange, purple, green, blue, black, and red respectively. The bottom panel shows which subclones each sample in G1 contains. Variants in C10 shared by all samples in G1 but not in Ln1, BrP and BrM. Sample Kd1, Ln4, Lv1 and Lv2 are clonal, whereas Lv4 and Pa1 had different subclones. Lv4 were polyclonal seeded by Pa1. **(B)** Estimated CP of each subclone of Pa1 and Lv4. Each blue circle represents an inferred subclone and each box represents a sample. **(C)** Migration pattern illustration for samples in G1.

Fig. S8

A

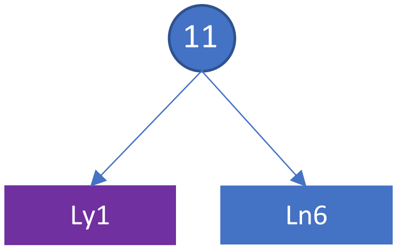

B

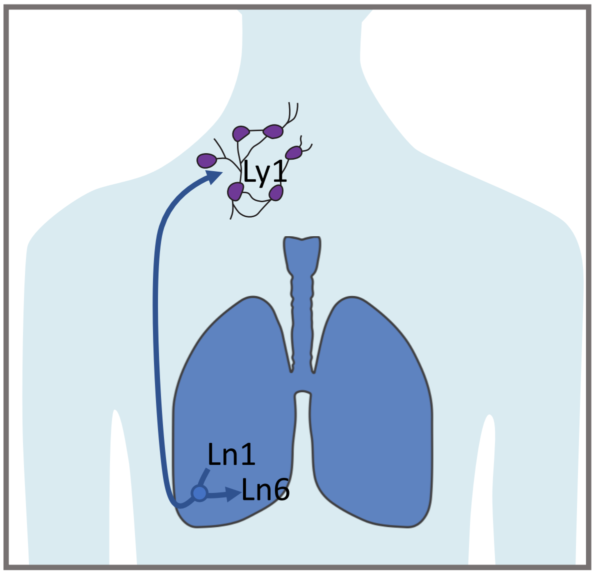

Fig. S8. Samples in G2, Ly1 and Ln6 were clonal. (A) G2 samples Ly1 and Ln6 were seeded by common ancestor Sc11. Each blue circle represents an inferred subclone and each box represents a sample. **(B)** Migration pattern illustration for samples in G2.

Fig. S9

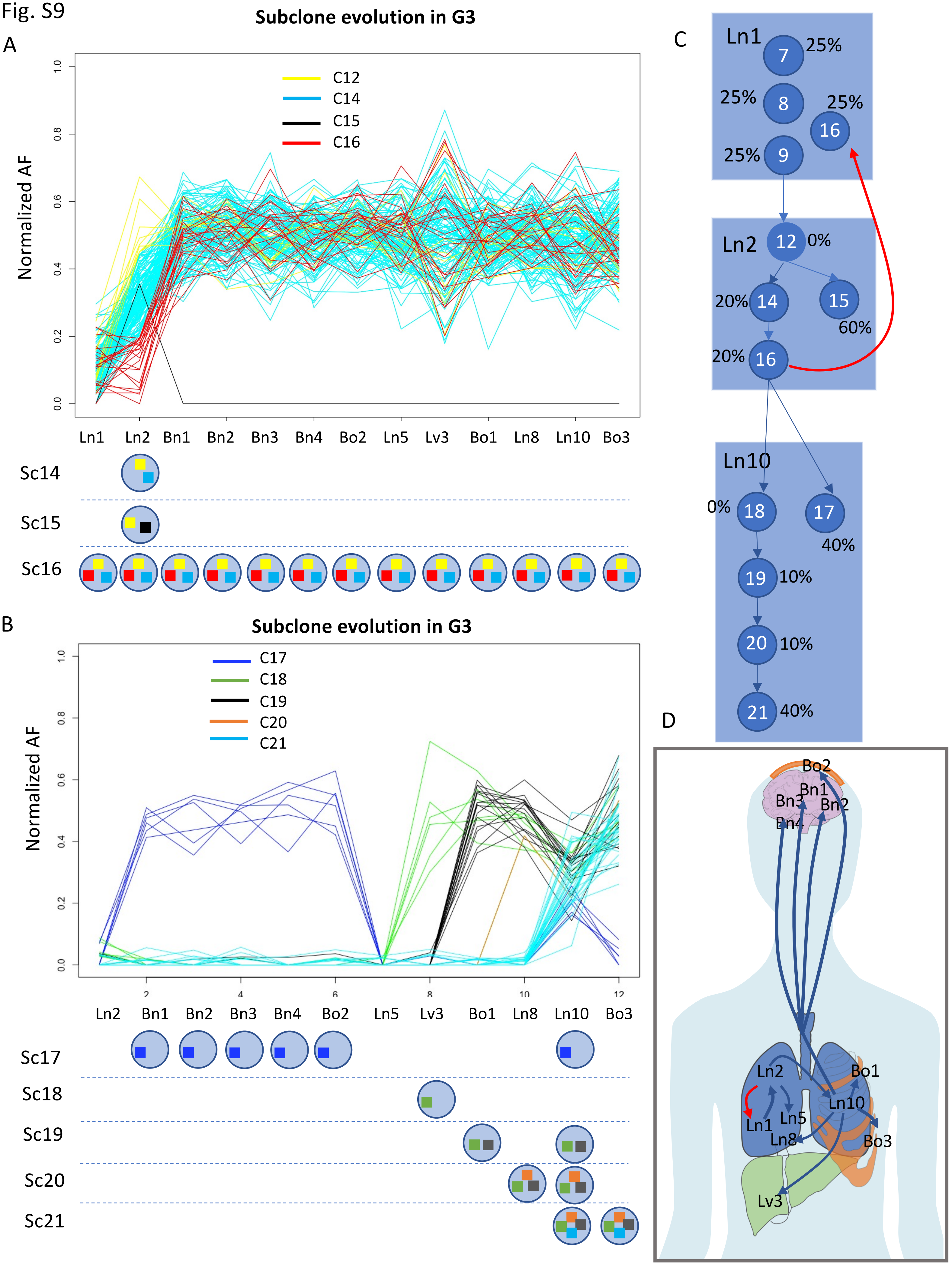

A
Subclone evolution in G3
Normalized AF
0.0
0.2
0.4
0.6
0.8
1.0
C12
C14
C15
C16
Ln1
Ln2
Bn1
Bn2
Bn3
Bn4
Bo2
Ln5
Lv3
Bo1
Ln8
Ln10
Bo3
Sc14
Sc15
Sc16
B
Subclone evolution in G3
Normalized AF
C17
C18
C19
C20
C21
2
4
6
8
10
12
Ln2
Bn1
Bn2
Bn3
Bn4
Bo2
Ln5
Lv3
Bo1
Ln8
Ln10
Bo3
Sc17
Sc18
Sc19
Sc20
Sc21
C
Ln1
7
25%
25%
8
25%
16
25%
9
Ln2
12
0%
20%
14
15
60%
20%
16
Ln10
0%
18
17
40%
19
10%
20
10%
21
40%
D
Bo2
Bn1
Bn3
Bn2
Bn4
Ln2
Bo1
Ln10
Ln1
Ln5
Ln8
Bo3
Lv3

Fig. S9. Subclone evolution and expansion for samples in G3. Normalized AF of G3 specific somatic short variants on chromosomes 2, 7, 9, 11, 14, 15, 16, 21, and 22. (A) The top panel is normalized AF of short variants clustered into C12, C14, C15, and C16 depicted in yellow, cyan, black, and red respectively. The bottom panel is a schematic plot of which sample contains which subclones. The gradient AF of C12, C14, and C16 in Ln2 suggested evolution process Sc12->Sc14->Sc16 in Ln2. Thus the Ln2 was the birth-place of Sc16. The evidence that Sc16 also presented in Ln1 means that Sc16 recolonized Ln1 (red arrow in C). **(B)** The top panel is normalized AF of short variants clustered into C17, C18, C19, C20, and C21 depicted in blue, green, black, orange, and cyan respectively. The bottom panel is a schematic plot of which sample contains which subclones. Four brain samples and Bo2 evolved from a common ancestor Sc17 which also presented in Ln10. Sc18-Sc21 each of which seeded Lv3, Bo1, Ln8 and Bo3 respectively evolved in Ln10 indicating that Ln10 served as an incubator. **(C)** Estimated CP of each subclone of Ln2 and Ln10. Each blue circle represents an inferred subclone and each box represents a sample. **(D)** Migration pattern illustration for samples in G3.

Fig. S10

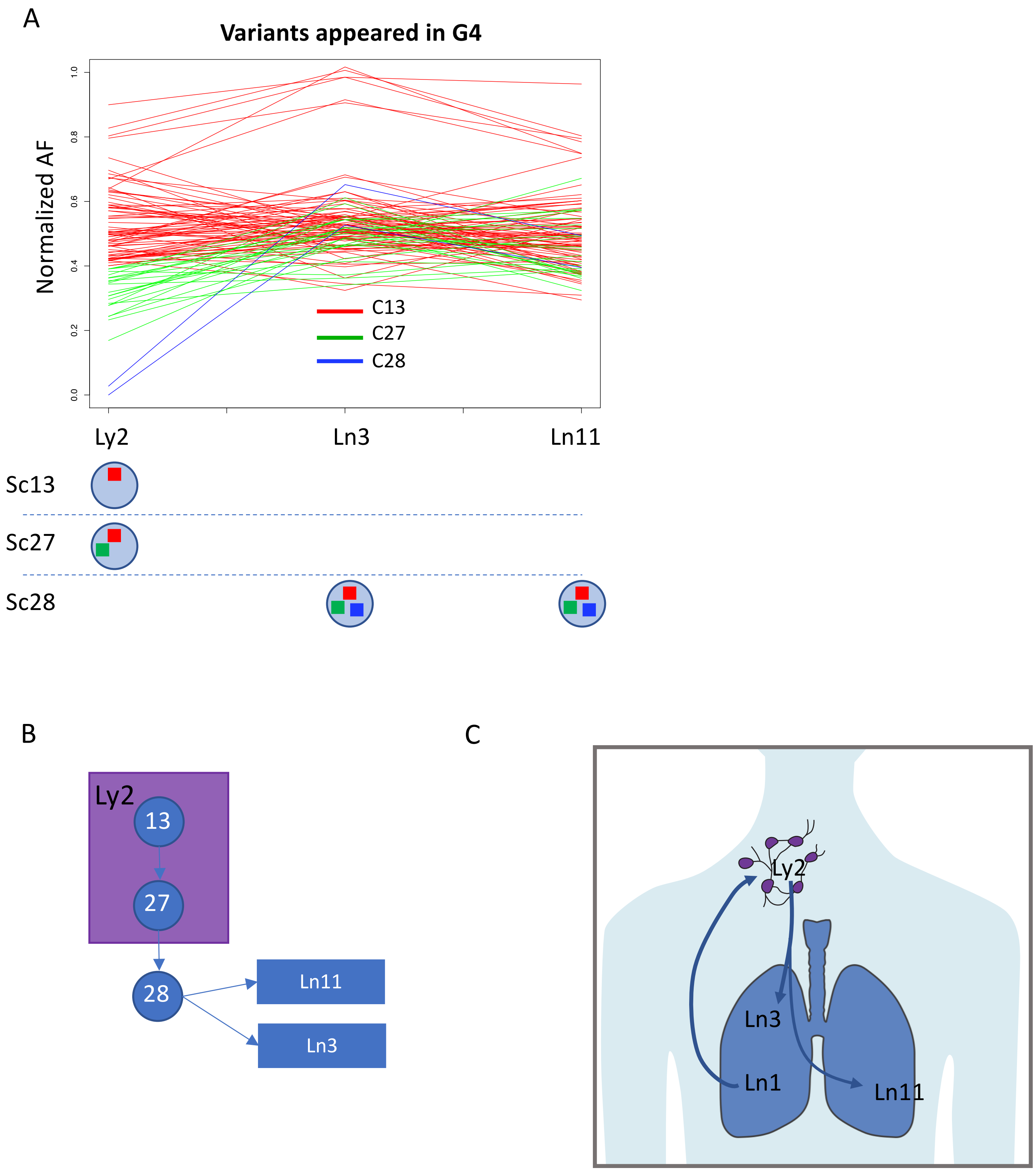
A
Variants appeared in G4
Normalized AF
1.0
0.8
0.6
0.4
0.2
0.0
C13
C27
C28
Ly2
Ln3
Ln11
Sc13
Sc27
Sc28
B
Ly2
13
27
28
Ln11
Ln3
C
Ly2
Ln3
Ln1
Ln11

Fig. S10. Subclone evolution and expansion for samples in G4. (A) The top panel is normalized AF of G4 specific somatic short variants on chromosome 2, 7, 9, 11, 14, 15, 16, 21, and 22 clustered into C12, C14, C15, and C16 depicted in yellow, cyan, black, and red respectively. The bottom panel is a schematic plot of which sample contains which subclones. **(B)** Reconstructed subclone structure for Ly2 reveals that Ln11 and Ln3 were seeded by a further evolved subclone Sc28 from Ly2. Each blue circle represents an inferred subclone and each box represents a sample. **(C)** Migration pattern illustration for samples in G4.

Fig. S11

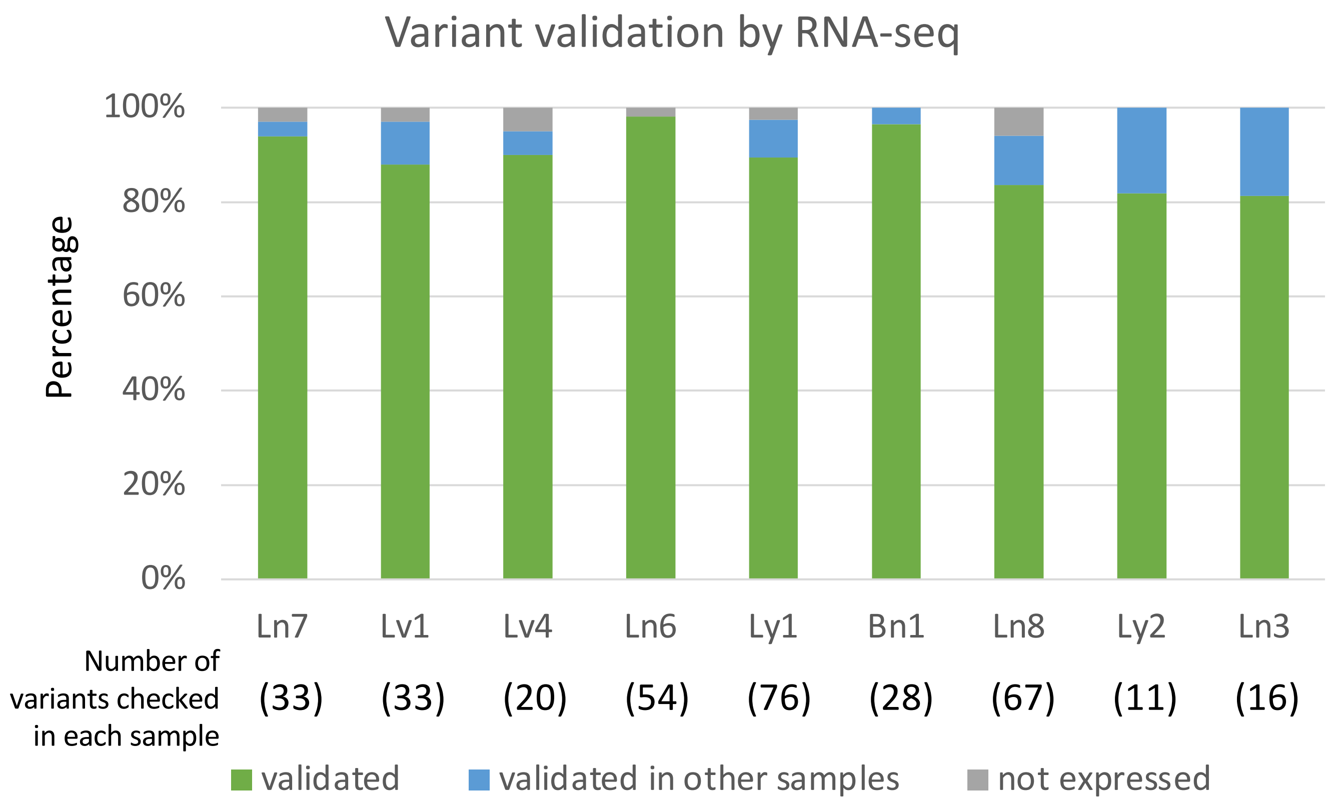

Variant validation by RNA-seq
Percentage
100%
80%
60%
40%
20%
0%
Ln7
Lv1
Lv4
Ln6
Ly1
Bn1
Ln8
Ly2
Ln3
Number of variants checked in each sample
(33)
(33)
(20)
(54)
(76)
(28)
(67)
(11)
(16)
validated
validated in other samples
not expressed

Fig. S11. Validation of somatic genomic short variants by RNA-seq data. Two samples randomly picked from each group as well as Ln7 were used for somatic genomic variant validation. For any given tumor sample, only somatic short variants that have greater than 10% genomic VAF, and have at least 10 reads in the paired RNA-seq were considered. The number of variants checked in each sample is shown under the sample name. Green portion represents the percentage of variants that were validated in paired RNA-seq data in each sample. Blue portion represents the percentage of the variants that were validated in RNA-seq data of other samples which share the same variants. Grey portion represents the variants that were not detected in either paired RNA-seq data or other samples' RNA-seq data. More than 90% of variants can be either "validated" or "validated in other samples".

Fig. S12

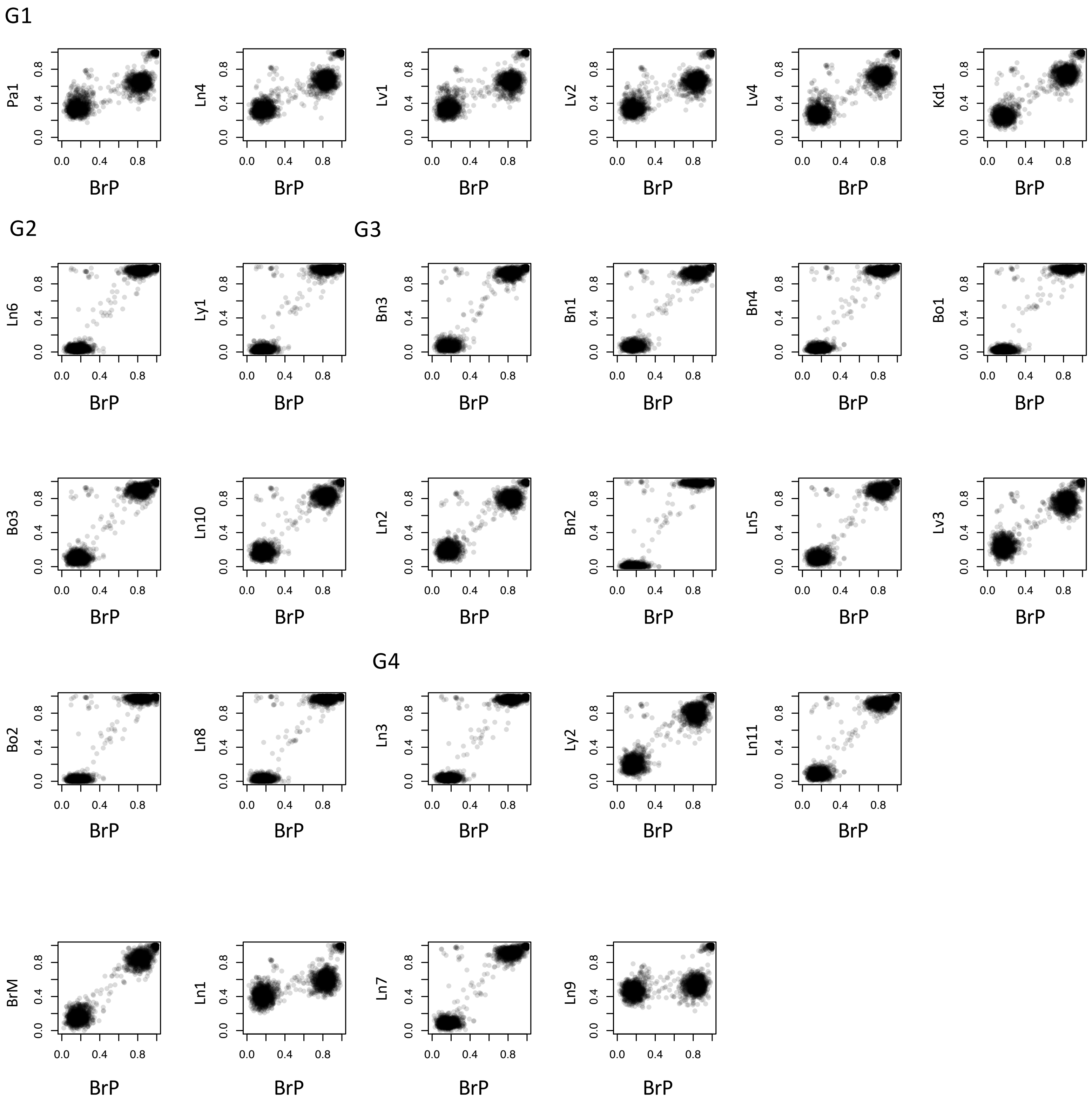
G1
Pa1
Ln4
Lv1
Lv2
Lv4
Kd1
G2
Ln6
Ly1
G3
Bn3
Bn1
Bn4
Bo1
Bo3
Ln10
Ln2
Bn2
Ln5
Lv3
Bo2
Ln8
G4
Ln3
Ly2
Ln11
BrM
Ln1
Ln7
Ln9
BrP
0.0
0.4
0.8

Fig. S12. Scatter plot of allele frequencies of inherited variants on chromosome 3 between BrP and other biopsies, to verify that the amplified allele in G1 was also the amplified allele in BrP and the unamplified allele was lost in G2, G3 and G4.

Fig. S13

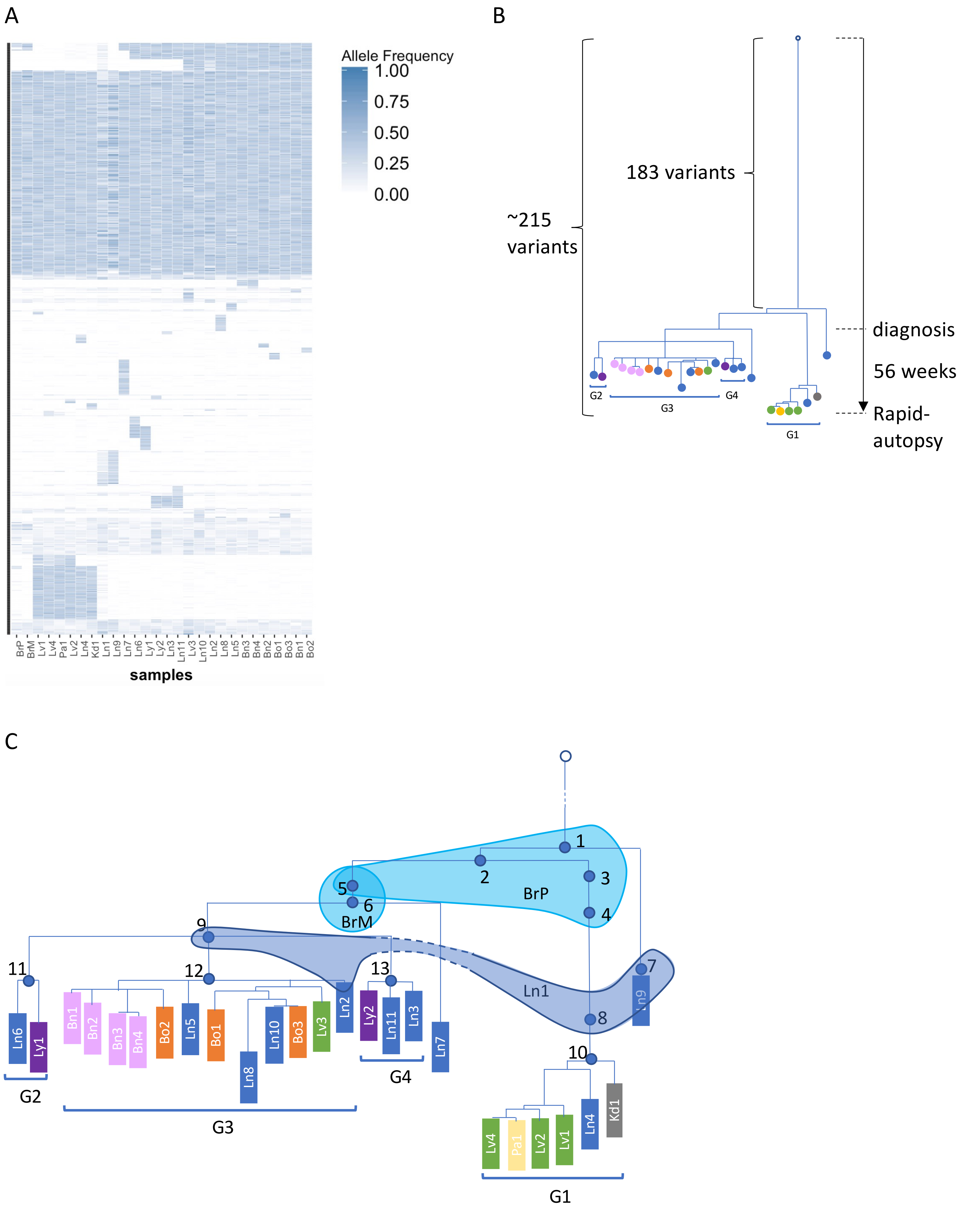
A
Allele Frequency
1.00
0.75
0.50
0.25
0.00
BrP BrM Lv1 Lv4 Pa1 Lv2 Ln4 Kd1 Ln1 Ln9 Ln7 Ln6 Ly1 Ly2 Ln3 Ln11 Lv3 Ln10 Ln2 Ln8 Ln5 Bn3 Bn4 Bn2 Bo1 Bo3 Bn1 Bo2
samples
B
183 variants
~215 variants
diagnosis
56 weeks
Rapid-autopsy
G2
G3
G4
G1
C
1
2
3
4
5
6
BrP
BrM
9
Ln1
7
8
10
11
12
13
Ln6
Ly1
Bn1
Bn2
Bn3
Bn4
Bo2
Ln5
Bo1
Ln8
Ln10
Bo3
Lv3
Ln2
Ly2
Ln11
Ln3
Ln7
Ln9
Lv4
Pa1
Lv2
Lv1
Ln4
Kd1
G2
G3
G4
G1

Fig. S13. Construction of the phylogenetic tree based on somatic short variants. (A) Allele frequencies of somatic variants on chromosome 14. Each row is a variant and each column is a sample. **(B)** Phylogenetic tree constructed by a perfect phylogeny algorithm using somatic variants on chromosome 14 considering shared and unique variants among samples. The sample order from the left to right is the same as in (C). The branch length was proportional to newly acquired mutations. There are 183 somatic variants before the first bifurcation whereas on average ~215 somatic short variants on each evolution path. The corresponding time scale over disease progression is shown on the right. **(C)** Revised phylogenetic tree for samples in groups by incorporating heterozygous short variants from chromosomes 2, 7, 9, 11, 14, 15, 16, 21 and 22 showing that LOH events happened in BrP. We placed the inferred subclones (blue dots labeled number 1-13) in ***Fig. 3B*** on this phylogenetic tree as their position on the evolution process. Three shades of blue containing different ancestral subclones (labeled BrP, BrM and Ln1) are the highly heterogeneous samples.

Fig. S14

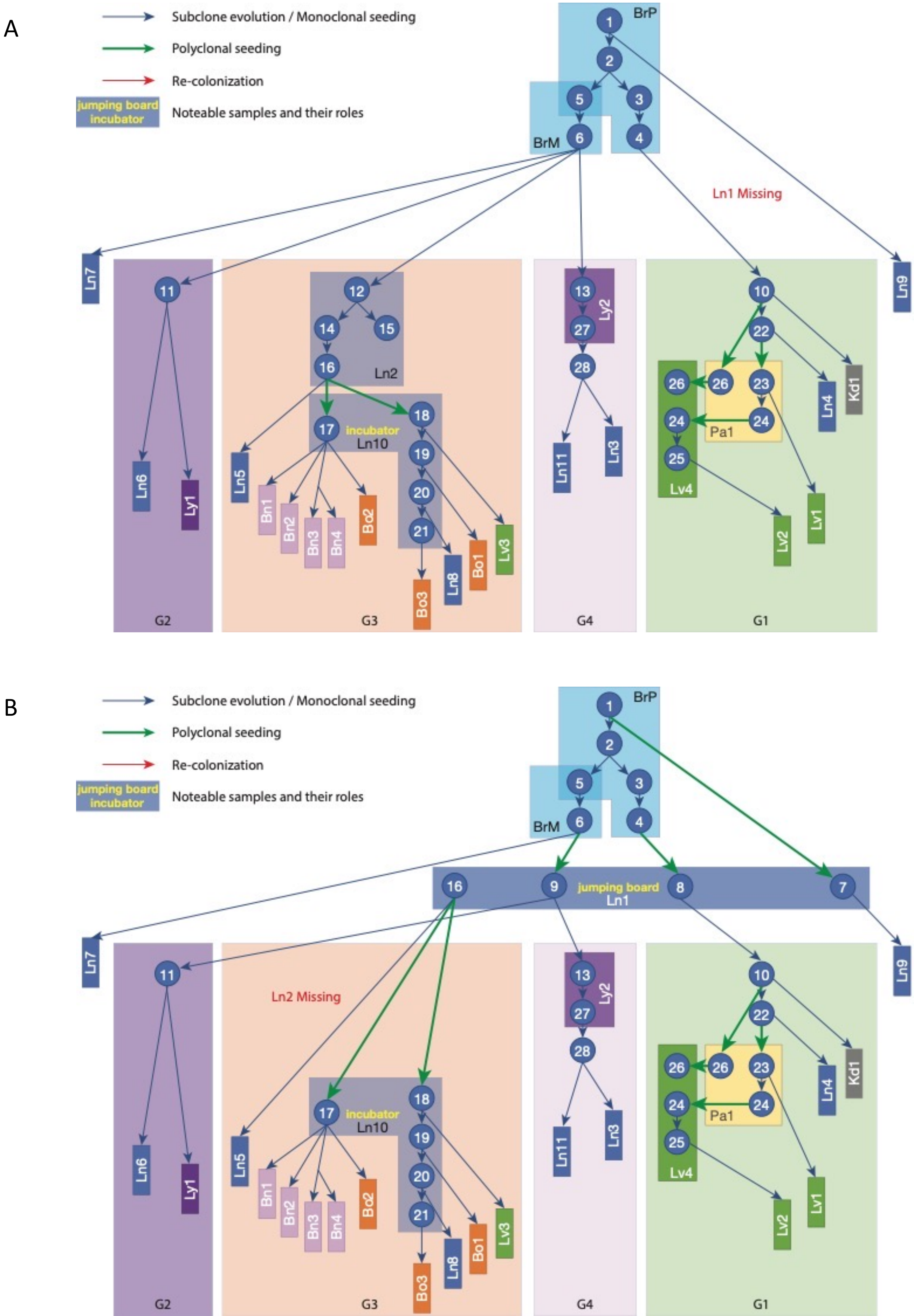
A
Subclone evolution / Monoclonal seeding
Polyclonal seeding
Re-colonization
jumping board
incubator
Noteable samples and their roles
BrP
BrM
Ln1 Missing
Ln7
Ln9
Ln2
incubator
Ln10
Ly2
Pa1
Lv4
Ln6
Ly1
Ln5
Bn1
Bn2
Bn3
Bn4
Bo2
Bo3
Ln8
Bo1
Lv3
Ln11
Ln3
Lv2
Lv1
Ln4
Kd1
G2
G3
G4
G1
B
Subclone evolution / Monoclonal seeding
Polyclonal seeding
Re-colonization
jumping board
incubator
Noteable samples and their roles
BrP
BrM
jumping board
Ln1
Ln2 Missing
incubator
Ln10
G2
G3
G4
G1

Fig. S14

C

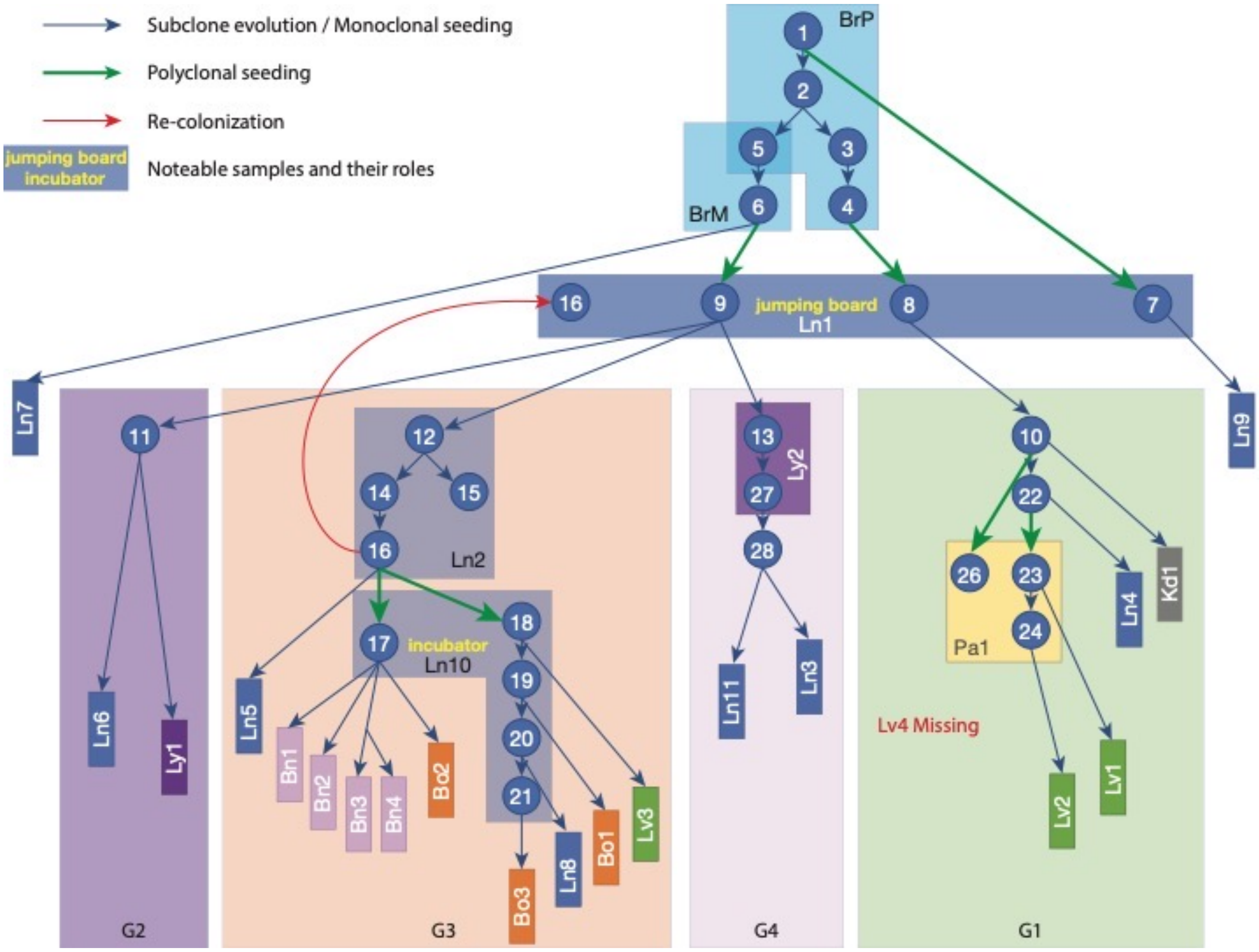

Subclone evolution / Monoclonal seeding
Polyclonal seeding
Re-colonization
jumping board
incubator
Noteable samples and their roles
BrP
BrM
jumping board
Ln1
Ln7
Ln9
Ln2
incubator
Ln10
Ln6
Ly1
Ln5
Bn1
Bn2
Bn3
Bn4
Bo2
Lv3
Bo1
Ln8
Bo3
Ly2
Ln11
Ln3
Pa1
Ln4
Kd1
Lv4 Missing
Lv2
Lv1
G2
G3
G4
G1

Fig. S14. Alternative subclonal expansion scenario with missing samples Ln1 (A), Ln2 (B), Lv4 (C).

Fig. S15

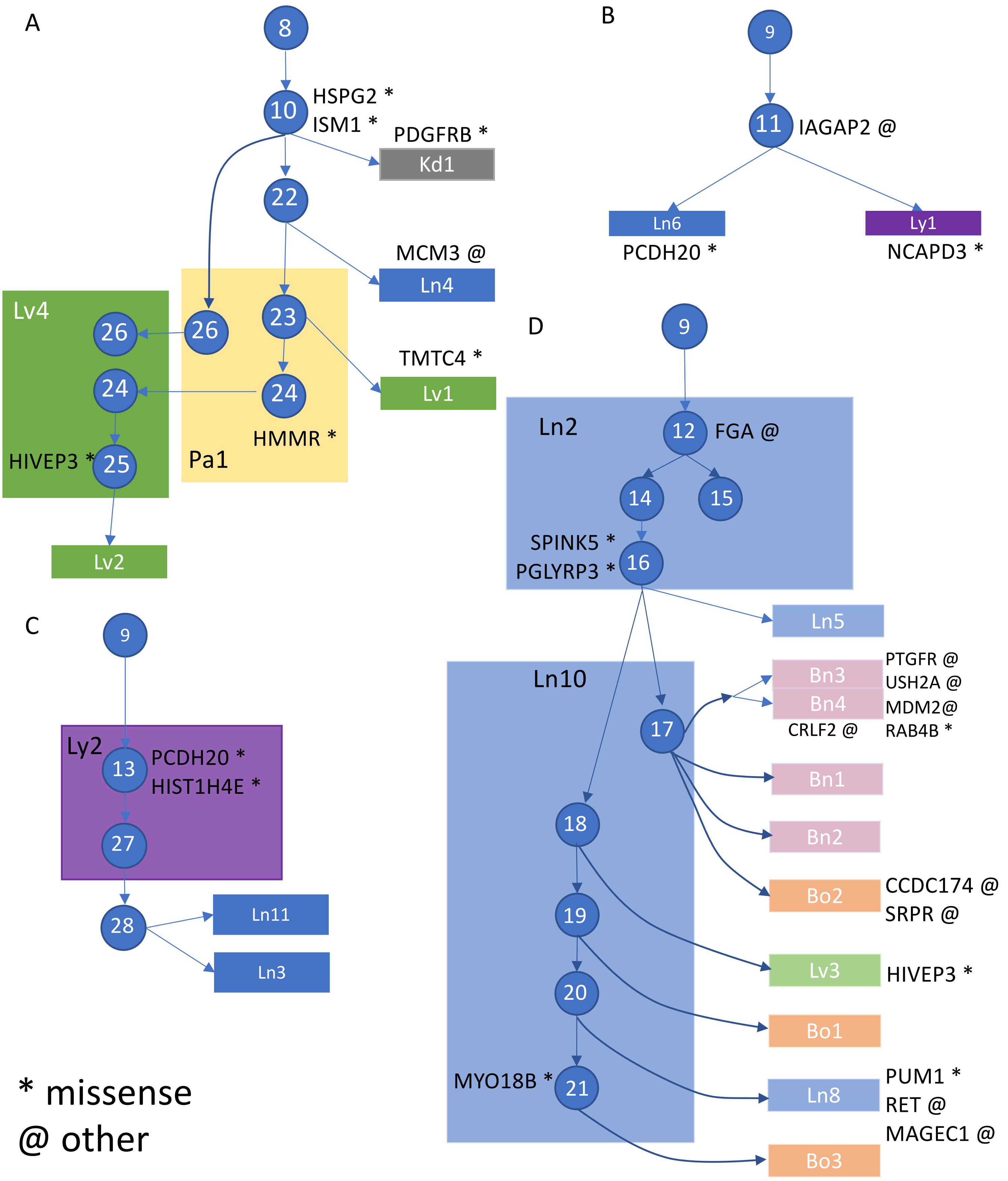
A
8
10
HSPG2 *
ISM1 *
PDGFRB *
Kd1
22
MCM3 @
Ln4
Lv4
26
26
23
TMTC4 *
Lv1
24
24
HMMR *
HIVEP3 *
25
Pa1
Lv2
B
9
11
IAGAP2 @
Ln6
PCDH20 *
Ly1
NCAPD3 *
C
9
Ly2
13
PCDH20 *
HIST1H4E *
27
28
Ln11
Ln3
D
9
Ln2
12
FGA @
14
15
SPINK5 *
PGLYRP3 *
16
Ln5
Ln10
17
Bn3
Bn4
PTGFR @
USH2A @
MDM2@
RAB4B *
CRLF2 @
Bn1
Bn2
18
Bo2
CCDC174 @
SRPR @
19
Lv3
HIVEP3 *
20
Bo1
MYO18B *
21
Ln8
PUM1 *
RET @
MAGEC1 @
Bo3
* missense
@ other

Fig. S15. Group or sample specific somatic coding variants (A) G1, (B) G2, (C) G4, and (D) G3. Missense mutations are depicted as *, and other coding mutations such as frameshift are depicted as @.

Fig. S16

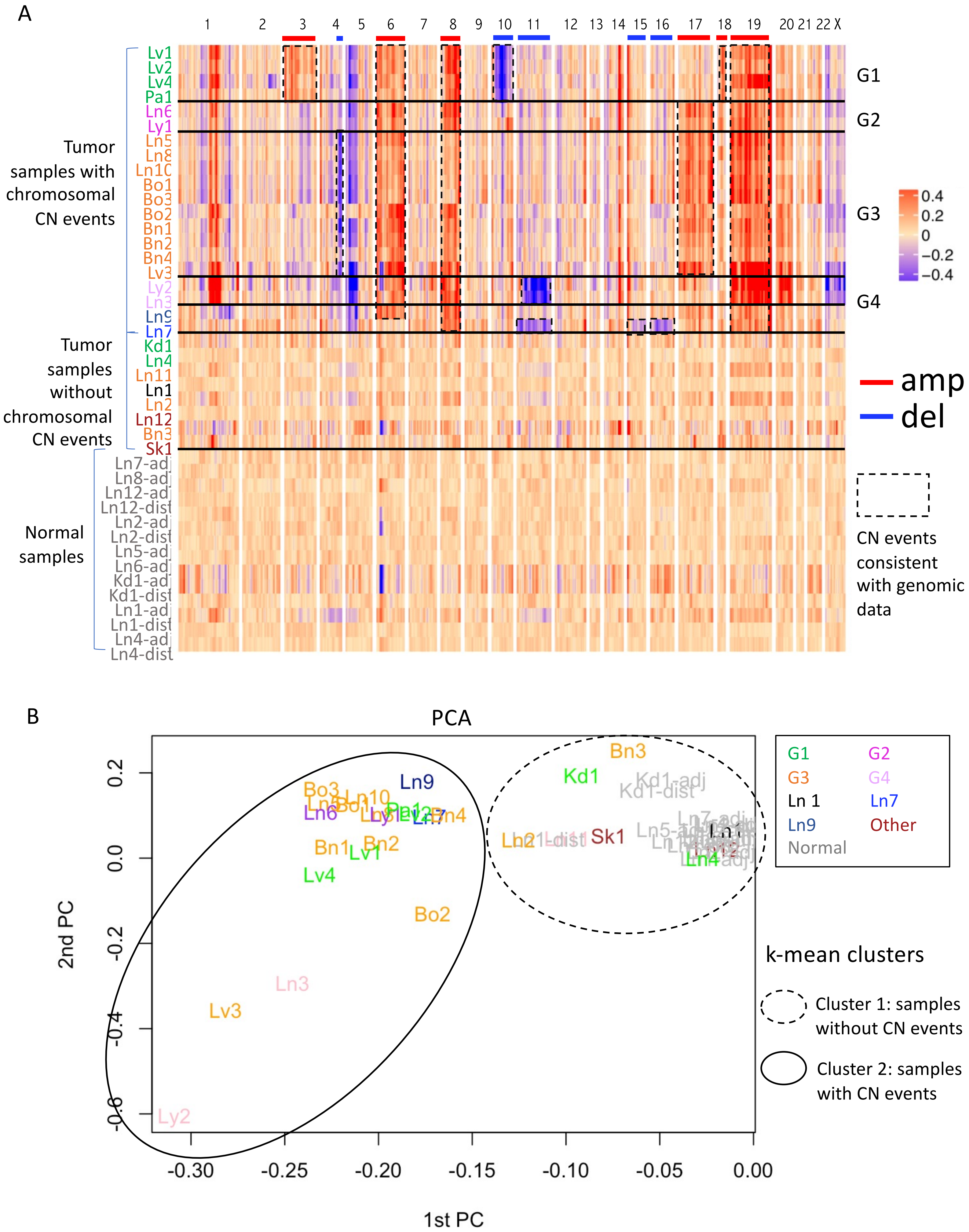
A
1 2 3 4 5 6 7 8 9 10 11 12 13 14 15 16 17 18 19 20 21 22 X
Lv1
Lv2
Lv4
Pa1
Ln6
Ly1
Ln5
Ln8
Ln10
Bo1
Bo3
Bo2
Bn1
Bn2
Bn4
Lv3
Ly2
Ln3
Ln9
Ln7
Kd1
Ln4
Ln11
Ln1
Ln2
Ln12
Bn3
Sk1
Ln7-adj
Ln8-adj
Ln12-adj
Ln12-dist
Ln2-adj
Ln2-dist
Ln5-adj
Ln6-adj
Kd1-adj
Kd1-dist
Ln1-adj
Ln1-dist
Ln4-adj
Ln4-dist
Tumor samples with chromosomal CN events
Tumor samples without chromosomal CN events
Normal samples
G1
G2
G3
G4
0.4
0.2
0
−0.2
−0.4
amp
del
CN events consistent with genomic data
B
PCA
1st PC
2nd PC
-0.30 -0.25 -0.20 -0.15 -0.10 -0.05 0.00
-0.6 -0.4 -0.2 0.0 0.2
G1 G2
G3 G4
Ln 1 Ln7
Ln9 Other
Normal
k-mean clusters
Cluster 1: samples without CN events
Cluster 2: samples with CN events

Fig. S16

C

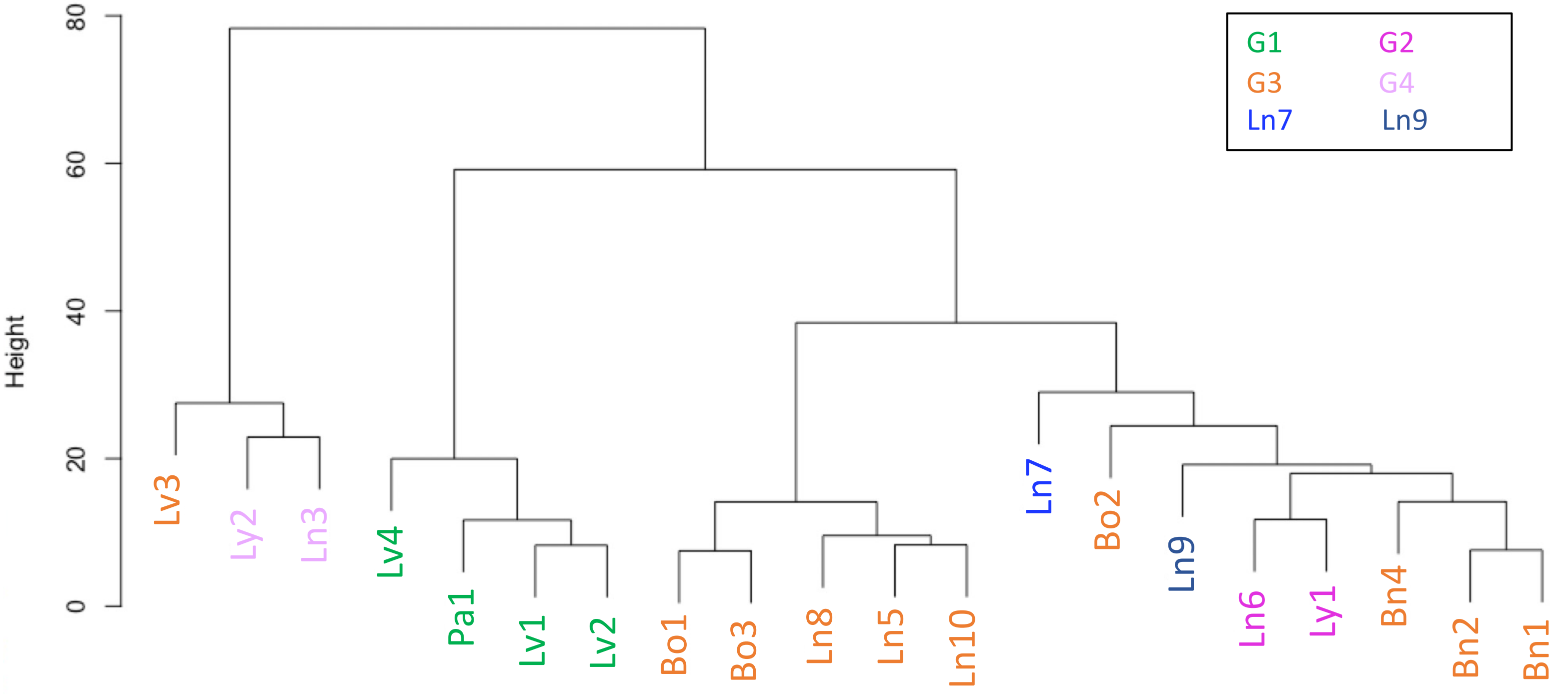

Height
80
60
40
20
0
G1
G2
G3
G4
Ln7
Ln9
Lv3
Ly2
Ln3
Lv4
Pa1
Lv1
Lv2
Bo1
Bo3
Ln8
Ln5
Ln10
Ln7
Bo2
Ln9
Ln6
Ly1
Bn4
Bn2
Bn1

Fig. S16. Copy number inferred from RNA sequencing data recapitulates genomic CNV events. (A) Copy number profiles inferred from the expression fold-change averaged across 101-gene windows for all samples. Twenty tumor samples from the top showed significant CN changes and recapitulated the presence of large CNVs from the WGS data. Major CN events from WGS data are highlighted in dotted box. Red and blue lines indicate amplification and deletion events respectively. **(B)** PCA analysis and unsupervised k-means clustering on the expression fold-change averaged across 101-gene windows showed that 8 tumor samples (Ln1, Ln2, Ln11, Ln4, Kd1 and Bn3) clustered together with normal samples indicating that they had heavy normal tissue contamination. **(C)** Hierarchical clustering of the twenty purest tumor samples showed that most samples in the same group defined by genomic data also clustered together.

Fig. S17

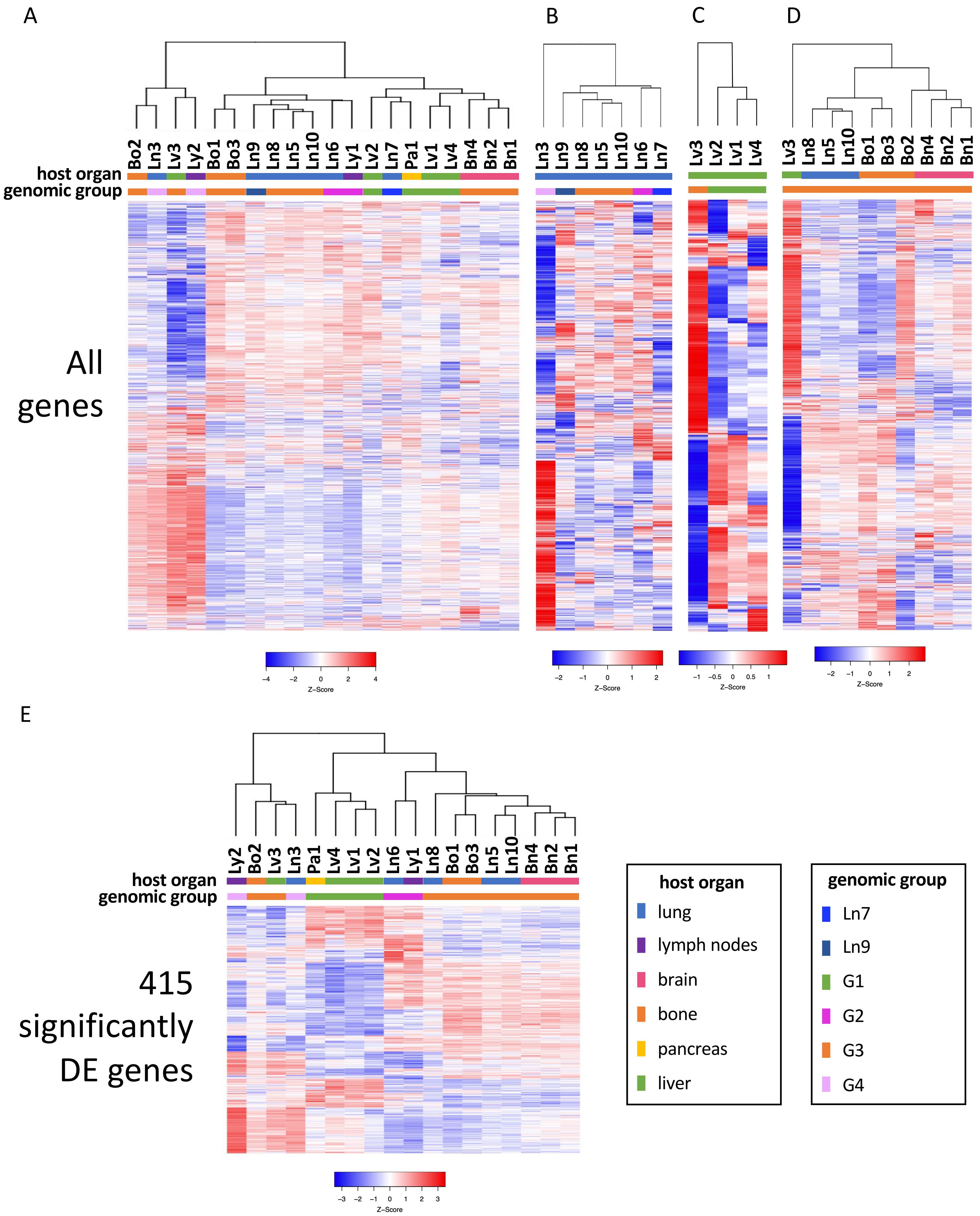
A
B
C
D
host organ
genomic group
Bo2 Ln3 Lv3 Ly2 Bo1 Bo3 Ln9 Ln8 Ln5 Ln10 Ln6 Ly1 Lv2 Ln7 Pa1 Lv1 Lv4 Bn4 Bn2 Bn1
Ln3 Ln9 Ln8 Ln5 Ln10 Ln6 Ln7
Lv3 Lv2 Lv1 Lv4
Lv3 Ln8 Ln5 Ln10 Bo1 Bo3 Bo2 Bn4 Bn2 Bn1
All genes
Z-Score
E
Ly2 Bo2 Lv3 Ln3 Pa1 Lv4 Lv1 Lv2 Ln6 Ly1 Ln8 Bo1 Bo3 Ln5 Ln10 Bn4 Bn2 Bn1
415 significantly DE genes
host organ
lung
lymph nodes
brain
bone
pancreas
liver
genomic group
Ln7
Ln9
G1
G2
G3
G4

Fig. S17. Transcriptomic profiling of metastatic tumors. (A) Heatmap of gene expressions across 20 metastatic samples of high tumor purity. (B) Heatmap of gene expressions of 7 lung metastatic samples. Unsupervised clustering showed that Ln5, Ln8 and Ln10, which all belong to G3, were more similar to each other than with Ln6 of G2 and Ln3 of G4. (C) Heatmap of gene expressions of 4 liver metastatic samples. Unsupervised clustering showed that Lv1, Lv2 and Lv4, which all belong to G1, were more similar to each other than to Lv3 in G3. (D) Heatmap of gene expressions of 10 samples in G3 which contained the most diverse host organ types. Unsupervised clustering showed that samples located in the same organ tend to cluster together. (E) Heatmap of 415 significantly differentially expressed genes across samples in G1, G2, G3 and G4.

Fig. S18

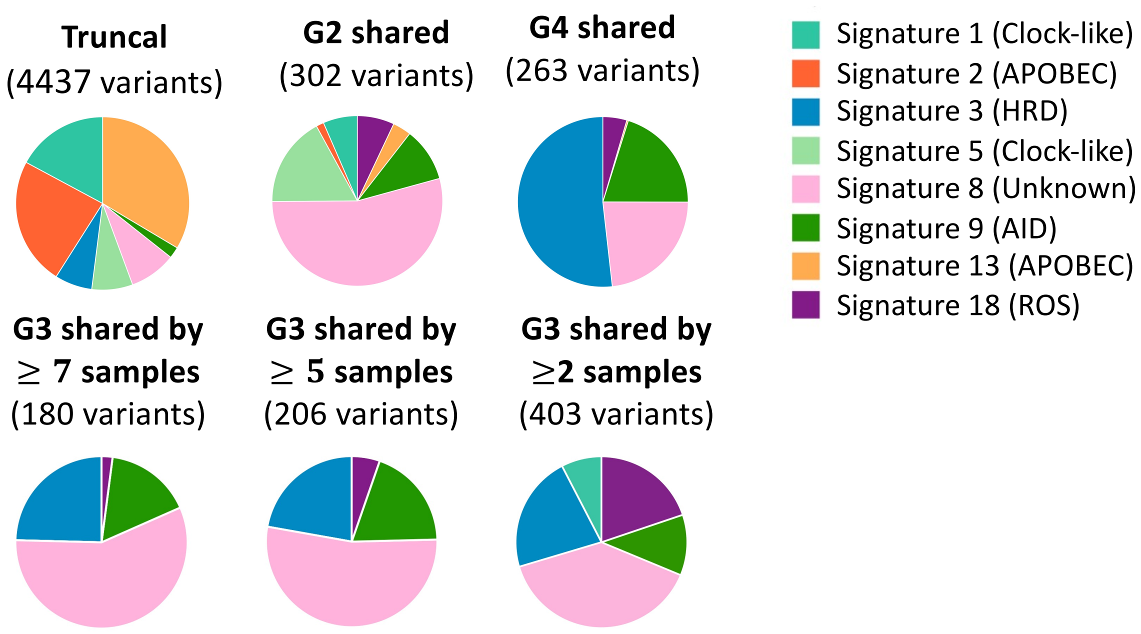

Truncal
(4437 variants)
G2 shared
(302 variants)
G4 shared
(263 variants)
G3 shared by
≥ 7 samples
(180 variants)
G3 shared by
≥ 5 samples
(206 variants)
G3 shared by
≥2 samples
(403 variants)
Signature 1 (Clock-like)
Signature 2 (APOBEC)
Signature 3 (HRD)
Signature 5 (Clock-like)
Signature 8 (Unknown)
Signature 9 (AID)
Signature 13 (APOBEC)
Signature 18 (ROS)

Fig. S18. Ancestral subclones of samples in G2, G3 and G4 evolved outside of the primary site. Pie chart of mutational signatures showed that signature 18 was present in G2, G3, G4 shared variants but not in the primary tumor samples (truncal).
