# Supplementary material for: Novel temporal and spatial patterns of metastatic colonization from breast cancer rapid-autopsy tumor biopsies
Source: Genome Med. 2021 Oct 28;13:170. doi: 10.1186/s13073-021-00989-6 (PMC8555066; doi:10.1186/s13073-021-00989-6)
Supplement: Supplementary file 3 — Additional file 3: Subclone analysis-alternative solutions. [file 13073_2021_989_MOESM3_ESM.docx]

**Additional File 3**

**Subclone analysis - alternative solutions**

From the phylogenetic tree (***Fig. 3A***), we already knew something about the structure of the tumor evolution trajectory in this patient: 1) the primary tumors and Ln1 are mixtures of the evolution lineage ancestors (***Fig. 3A***); and 2) samples in each group (G1-G4) have their own evolution lineages. Based on this information, in order to reduce computational complexity, we separated subclonal reconstruction into four parts: 1) reconstruction of early subclonal evolution (before group lineage separation); 2) reconstruction of subclonal evolution in G1; 3) reconstruction of subclonal evolution in G3; 4) reconstruction of subclonal evolution in G4. For two samples in G2, because they both have clonal shared and unique variants, they were likely the results of monoclonal seeding from a common ancestral subclone. Therefore, the reconstruction of the subclone structure for G2 is straightforward (***Fig. 4***).

SubcloneSeeker V2 produced 2 solutions for early subclonal evolution (***Figure A*** and ***B*** below). The differences between these two solutions are the placement of Sc4 and Sc8 (i.e., Sc4->Sc8 vs. Sc8->Sc4). Sc4 defining variants C4 have higher allele frequency than Sc8 defining variants C8 in BrP. In solution A which contains Sc4->Sc8, the cell prevalence (CP) of Sc4 and Sc8 are positive values, whereas in solution B which contains Sc8->Sc4, the CP of Sc8 in BrP is -0.14. Although Sc8->Sc4 is an algorithmical viable solution (CP error margin is ±0.2), Sc4->Sc8 is more parsimonious since it did not invoke negative cellular prevalence. Therefore, we used solution A in the manuscript (***Fig. 4***).

SubcloneSeeker V2 produced 2 solutions to G3 subclone reconstruction (***Figure C*** and ***D*** below). The differences between these two solutions are the placement of Sc15 (i.e., Sc12->Sc15 vs. Sc9 ->Sc15). The placement of Sc15 only affects sample Ln2 because Sc15 is an Ln2 unique subclone. The CP for Sc12 and Sc15 in Ln2 are 0.64 and 0.42. Just based on this, they can be either of a parent-child relationship or two children of a common parent Sc9. However, orthogonal analysis from CNV estimated that Ln2 had 60% tumor content. After we correct the CP for tumor purity, the CP of Sc12 and Sc15 in Ln2 become 1.06 and 0.7. The parent-child relationship of Sc12 and Sc15 becomes the only solution. Therefore, we used solution C in the manuscript (***Fig. 4***).

SubcloneSeeker V2 produced unique solutions for G1 (***Figure E***) and G4 (***Figure F***) subclone reconstructions respectively, which are shown in the manuscript (***Fig. 4***).


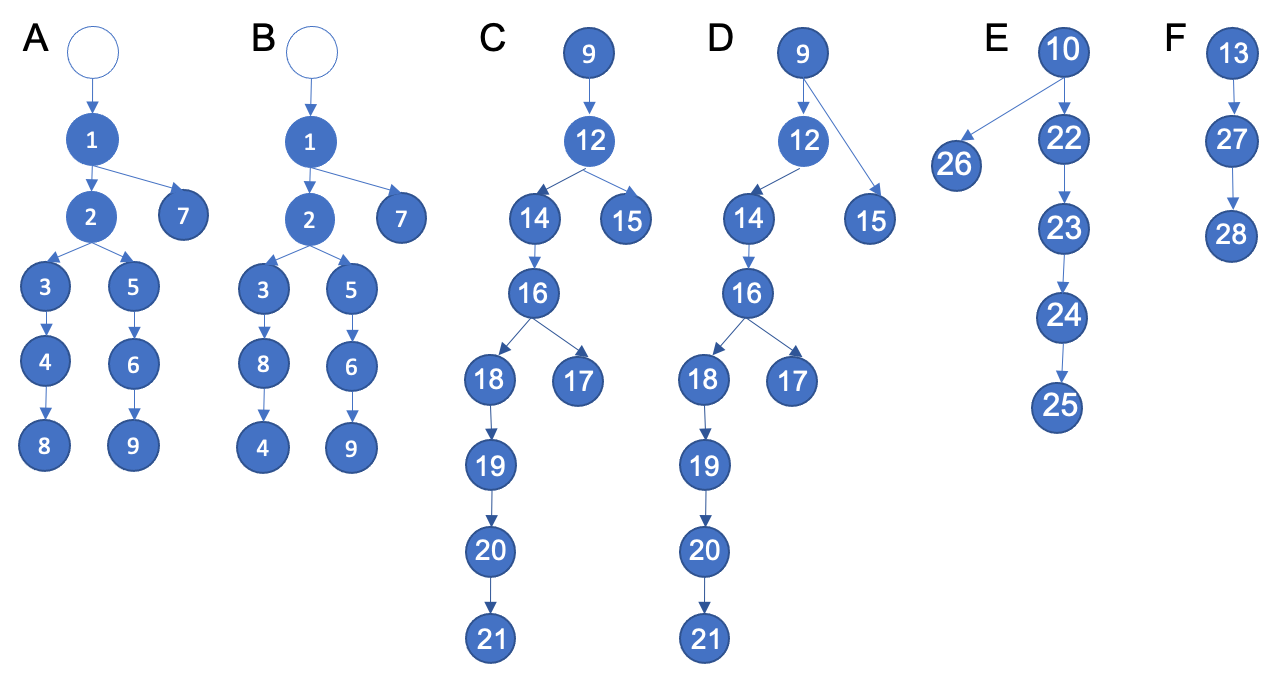


**Subclone reconstruction solutions generated by SubcloneSeeker V2. (A, B)** Solutions for early subclone evolution. **(C, D)** Solutions for G3 subclone reconstruction. (**E**) Unique solution for G1 subclone reconstruction. (**F**) Unique solution for G4 subclone reconstruction.
